# Population preferences for non-pharmaceutical interventions to control the SARS-CoV-2 pandemic: trade-offs among public health, individual rights, and economics

**DOI:** 10.1007/s10198-022-01438-w

**Published:** 2022-02-09

**Authors:** Axel C. Mühlbacher, Andrew Sadler, Yvonne Jordan

**Affiliations:** 1 Gesundheitsökonomie und Medizinmanagement, Hochschule Neubrandenburg, Brodaer Straße 2, 17033 Neubrandenburg, Germany; 2 Gesellschaft Für Empirische Beratung GmbH, Freiburg, Germany; 3 Duke Department of Population Health Sciences and Duke Global Health Institute, Duke University, Durham, NC USA

**Keywords:** SARS-CoV-2, Population preference, Discrete choice experiments, Best–worst scaling, C2, C23, C35, I12, I18, D79

## Abstract

**Problem:**

Policymakers must decide on interventions to control the pandemic. These decisions are driven by weighing the risks and benefits of various non-pharmaceutical intervention alternatives. Due to the nature of the pandemic, these decisions are not based on sufficient evidence regarding the effects, nor are decision-makers informed about the willingness of populations to accept the economic and health risks associated with different policy options. This empirical study seeks to reduce uncertainty by measuring population preferences for non-pharmaceutical interventions.

**Methods:**

An online-based discrete choice experiment (DCE) was conducted to elicit population preferences. Respondents were asked to choose between three pandemic scenarios with different interventions and impacts of the Corona pandemic. In addition, Best–worst scaling (BWS) was used to analyze the impact of the duration of individual interventions on people’s acceptance. The marginal rate of substitution was applied to estimate willingness-to-accept (WTA) for each intervention and effect by risk of infection.

**Results:**

Data from 3006 respondents were included in the analysis. The DCE showed, economic effect of non-pharmaceutical measures had a large impact on choice decisions for or against specific lockdown scenarios. Individual income decreases had the most impact. Excess mortality and individual risk of infection were also important factors influencing choice decisions. Curfews, contact restrictions, facility closures, personal data transmissions, and mandatory masking in public had a lesser impact. However, significant standard deviations in the random parameter logit model (RPL) indicated heterogeneities in the study population. The BWS results showed that short-term restrictions were more likely to be accepted than long-term restrictions. According to WTA estimates, people would be willing to accept a greater risk of infection to avoid loss of income.

**Discussion:**

The results can be used to determine which consequences of pandemic measures would be more severe for the population. For example, the results show that citizens want to limit the decline in individual income during pandemic measures. Participation in preference studies can also inform citizens about potential tradeoffs that decision-makers face in current and future decisions during a pandemic. Knowledge of the population’s preferences will help inform decisions that consider people’s perspectives and expectations for the future.

Survey results can inform decision-makers about the extent to which the population is willing to accept certain lockdown measures, such as curfews, contact restrictions, lockdowns, or mandatory masks.

**Supplementary Information:**

The online version of this article (10.1007/s10198-022-01438-w) contains supplementary material, which is available to authorized users.

## Introduction

After the outbreak of SARS-CoV-2 (Corona virus), contact and exit restrictions, distance between people, hygiene rules, mandatory masking, and other interventions were introduced to protect public health and prevent overburdening the health care system. These measures have an impact on economic, social, and cultural life [1–3]. Lockdowns have been conducted repeatedly over several weeks, and in some cases have transitioned to tighter lockdown. Nevertheless, rising infection rates have been reported. A temporary recovery in infection rates was followed by further tightening with business and school closures, hard lockdowns with curfews, a contact restrictions that remained in effect even over holidays. Loosening and tightening measures in constant change become tiring for the population in the long run, especially if further drastic measures do not bring the intended success and there is no end to normality on the horizon. Therefore, it is important to know the preferences of the population and to consider them when implementing measures. Despite various studies, there is currently a lack of structured elicitation, measurement, and analysis of population preferences for pandemic measures.

Policymakers must balance health and life, privacy, and the maintenance of economic, social, cultural, and civic life. It remains unclear to what extent citizens support and accept the measures. Therefore, comprehensive and reliable information is needed to avoid rash decisions and to maintain the right balance in the implementation and necessary maintenance of measures. The acceptance of measures depends to a large extent on whether the preferences of those mainly affected by these measures are taken into account. Only with knowledge of the preferences can decisions be made which, in perspective, meet people’s expectations [2, 4, 5]. The time component plays a particularly important role here. How long will restrictive measures be accepted? Which measures are accepted by the citizens in the short term, and which in the long term?

Many interesting research studies were conducted during the pandemic. The studies varied from vaccine preferences [6–15] to the acceptance of artificial intelligence [16], and digital contact tracing [17, 18]. However, there are few studies on preferences and acceptability of non-pharmaceutical interventions [19–21]. Chorus et al. [19] examined the willingness of Dutch society to accept or sacrifice deaths to avoid physical, psychological, educational, and economic effects of lockdowns. Their results suggest that the average citizen would be willing to accept substantial losses, such as a delay in educational achievement or a permanent and substantial reduction in net incomes. However, the willingness to accept making sacrifices varies greatly among population groups. A study by Ozdemir et al. [20], which is one of the first studies to examine the likelihood of support for government action in response to different infectious disease outbreaks, used hypothetical vignettes instead of a DCE. The results showed that the likelihood of support for a given intervention is highly dependent on characteristics of the outbreak, such as the number of new cases within the past 2 weeks. Reed et al. [21] used a DCE and a ranking exercise to examine the extent to which U.S. citizens would be willing to accept greater spread of SARS-CoV-2 in exchange for lifting restrictions that affect social and economic life. Their results show that respondents consider the reopening of non-essential stores to be most important. In addition, the results suggest that political affiliation does not have a strong impact on attitudes toward socially distancing policies. People’s willingness to accept trade-offs between health and non-health outcomes is not related to political affiliation.

The aim of this empirical study is to evaluate alternative non-pharmaceutical interventions and determine the value of such interventions from a population perspective. In contrast to existing studies, we use a more comprehensive decision model that equally considers pandemic measures and potential impacts at different levels, such as society and the economy. Ultimately, we aim to generate evidence for better decision-making, not only in times of SARS-CoV-2 pandemic, by capturing the benefit and risk perceptions of the interventions and the associated preferences of the population. Here, the main questions are: What constitutes value and therefore impacts the acceptance of non-pharmaceutical interventions? What trade-offs are people willing to make, e.g., when it comes to personal or public financial losses, loss of life, restriction of personal freedom and individual rights?

## Methods

In this stated preference study, two stated preference methods are applied, a Discrete Choice Experiment (DCE) and a Best–Worst Scaling (BWS). Both, the DCE and BWS are recognized preference measurement instruments. They have been used to measure and analyze patient preferences in health care and, also to support policy decisions. Stated preference methods are useful when data on observable preferences are not available. These methods were developed to analyze information about an individual’s willingness to accept trade-offs between individual attributes of a product or service. A DCE, for example, is based on the hedonistic principle that an intervention can be described by various attributes and that the acceptance or attractiveness of an alternative is a function of these attributes. DCEs and BWS assume that individuals choose an option which offers the highest (relative) benefit.[22–27]. DCEs are widely accepted in the health care system and are increasingly being discussed by regulatory authorities [28]. The study followed accepted guidelines [22, 23, 26].

### Discrete choice experiment

A DCE was designed to elicit the preferences of the population in Germany for non-pharmaceutical pandemic interventions. The method is particularly appropriate when decision-making is characterized by trade-offs. The trade-offs faced by decision-makers and potential consequences faced by citizens can be comprehensibly analyzed. It can be quantitatively measured which attributes citizens consider important compared to other attributes and how citizens weigh and trade off different attributes, such as impact and consequences of policy decisions in a pandemic. In a DCE-survey, respondents are asked to choose among discrete alternatives based on their preferences and to choose the best alternative and, in special cases, the worst alternative. The alternatives in this study are lockdowns scenarios characterized by different attributes.

### Attributes and levels

DCEs assume that a health product or service can be described by attributes and attribute levels [27]. Attributes are decision criteria that influence the choice of the preferred alternative. The attributes used in the present study were identified by a nonsystematic literature search that included newspaper articles, initial research reports, and internet articles about previous pandemics. The attributes in the present study represent economic and social measures taken by political decision-makers (e.g., contact restrictions [1, 3, 20, 29–31], exit restrictions [1, 20, 30, 31], closure of businesses and facilities [1, 3, 4, 20, 21, 29–31], mask obligation [3, 4, 29] and data transmission/digital tracking [4, 18, 32]) and possible consequences of these measures (e.g., decline in the country’s economic performance or individual income [2, 19]). Other attributes relate to consequences of the coronavirus itself (e.g., excess mortality [2, 19] or risk of infection [21]).

Table 1 lists all attributes used in the DCE that describe measures and possible effects of the measures. Each attribute is described by different levels that indicate the extent to which measures are implemented, e.g., closing national borders, or impacting individual economic levels, e.g., 10% decrease of annual individual income. A complete list of all attributes and levels used in the DCE can be found in the ESM Appendix.

**Table 1:** Characteristics of non-pharmaceutical interventions

		
Excess mortality	Individual risk of infection	Economic performance
		
Individual income	Curfews	Contact restrictions
		
Closures	Personal data	Mask obligation

### Best–worst scaling case 2

A special form of classical DCE is best–worst scaling (BWS). BWS tasks can be divided into three different cases: “object” (case 1), “profile” (case 2) and “multiprofile” (case 3) [33].

In the BWS case 2 (profile case), the choice set has the structure of a single profile and shows the same attributes in each scenario, while their levels change. The respondents do not have to consider the overall profile’s value, but needs to consider the attribute levels it defines, and choose both the best (most preferred) and the worst (least preferred) attribute levels in each presented scenario. The attribute levels are only part of an overall profile and thus there is no explicit choice trade-off per se [34, 35]. In this study, a mixture of BWS case 1 and 2 was used to examine the most and least preferred measures in terms of contact and exit restrictions and facility closures over different time periods (2, 4, or 12 weeks). The mixture of case 1 and 2 allowed multiple levels of an attribute to occur in a set. This made direct comparisons between different levels within an attribute possible, such as the closure of kindergartens and schools. Each respondent was presented with 11 choice sets which each consisted of 5 attribute levels (item). Respondents choose the best (most preferred) and the worst item (least preferred) from each choice set.

### Partial profile design

The experimental design consisted of 100 versions (blocks). Each respondent was assigned to 1 of the 300 blocks and answered 12 choice tasks each with three pandemic scenarios. Respondents were asked to choose the most preferred pandemic scenario. A dominance test was added as additional choice task to assess validity (rationality) of respondents’ choice decisions. One scenario in the dominance test had objectively clearly better attributes in terms of excess mortality, risk of infection, and decrease in income.

Respondents sometimes make choice only based on one dominant attribute rather than making a trade-off among all attributes. Especially in preference studies with many attributes, respondents may become overwhelmed by the complexity of the choice tasks and make choices that are not fully compensatory [36]. To prevent fatigue, it is recommended to keep a subset of attributes constant in each choice tasks. Constant attributes are then alternately hidden or grayed out so that the remaining attributes form the choice set to be evaluated. The corresponding experimental designs are referred to as partial profile designs [24, 36]. In this study, given the extensive decision model with the multitude of attributes and levels, a partial profile design was used. Each choice task contained a subset of the attributes, which means that for each scenario, 4 out of 9 attributes were shown to the respondents in different arrangements. Additionally, attributes were randomized to control for order effects across respondents.

The actual choice tasks were preceded by a graphical example. In the example, a choice task was presented and explained in detail. Respondents were asked to assume that the coronavirus pandemic and the established measures have different economic and social impacts. They were asked to think about different decision scenarios and choose the best scenario from their point of view. They were advised that there are no right or wrong answers and the scenarios available for choice do not have to correspond to reality in this form. The dominance test was used as warm-up task.

### Scope test

One criticism of stated preference analysis is that the results are not as sensitive to differences in the size or scope of the valued good as would be expected from economic theory [37].

A scope test can be used to analyze respondents’ sensitivity to the scope of an attribute level. This determines how differences in magnitude affect a willingness to pay (WTP) or a willingness to accept (WTA) certain risks. In a scope test, the same good is presented to the participants in different scales and it is analyzed whether a WTP or, as in the present study, WTA changes [38]. In the present study, we conducted a split sample with two survey versions. One of the two versions included a scope test for the attributes of excess mortality, individual risk of infection, decline in GDP, and decline in annual income. The last level of each of the four attributes was used in its magnitude. For example, the last level of the attribute risk of infection in questionnaire 1 contained the value 25% and in the scope test of questionnaire 2 the value 35%. This was used to examine whether respondents were sensitive to scale differences and, for example, how sensitive their willingness to accept risk was to scale differences. Respondent were randomly assigned to one of the versions.

The analysis of scope sensitivity was not the focus of this paper, but outlining the methodological approach contributes to a better understanding of the results. The results presented in this paper are based on an aggregated model of both questionnaire versions. Results for the scope test are provided in the ESM Appendix.

### Statistical analysis

A random parameter logit model RPL, also mixed logit model, assumes that the probability of choosing an alternative from a set of alternatives is a function of attribute levels and a random error term that accounts for individual-specific variation in preferences. RPL assumes that there is a distribution of preference weights, reflecting differences in respondents’ preferences, across the sample. An RPL models the parameters of this distribution for each attribute level. As a result, a mean effect as well as a standard deviation of the effects across the sample is obtained [23]. The BWS was analyzed using frequency analysis [39].

### Recruitment

The survey was conducted from October to November 2020. Recruitment of respondents was realized in cooperation with the market research company Dynata. Respondents were screened by gender, age, education, and region. Respondents aged ≥ 18 years, with good or very good German language skills.  To reflect the population in certain characteristics, systematic sampling was applied (quota sampling). Prior to participation, patients gave informed consent. Participation was voluntary and anonymous.

The survey period was in the middle of the second wave of the pandemic, when incidences increased rapidly. The delta variant (B.1.617.2), first detected in India in October 2020, dominated the pandemic in many countries at that time, including Germany. This led to renewed restrictions on public life, especially in the areas of culture and leisure. Vaccinations were not yet approved.

## Results

In total, 3467 respondents completed the online questionnaire. We created an exclusion index composed of completion time, trade-offs in the choice experiment, and control questions. Respondents who took less than 6 min to complete the survey were excluded (after adjusting for outliers mean completion time was about 20 min with a standard deviation of 10). Also excluded were respondents who made no trade-offs in choice decisions and answered simple control questions incorrectly. The exclusion resulted in improved model quality criteria such as the Akaike Information Criterion (AIC). As a result, 461 respondents were excluded from the analysis. A total of 3006 respondents were eventually included in the analysis. The choice probabilities for the three alternatives in the DCE were 31.86%, 35.97%, and 32.17%.

### Characteristics of the respondents

At 53.79 percent, there were slightly more women in the sample. The mean age was 51 years. The majority of respondents (57.29%) had a medium educational status, e.g., secondary school. About one-third of the respondents (34.66%) were employed full-time, and another third were retirees or pensioners (33.17%). A small proportion of respondents (13.87%) reported working in system-related jobs, which include jobs providing public safety and infrastructure (e.g., police officers, firefighters, postal workers, water supply workers, waste management workers), daily needs jobs (e.g., grocery stores, drug stores), health care workers (e.g., medical workers, dentists, veterinarians). Almost half of the respondents were married or in a registered partnership (47.21%). A more detailed overview of sociodemographic data of the respondents can be found in Table 2.

**Table 2:** Respondent characteristics

Characteristic	Mean ± std. dev	*N* = 3006	%
Age (years)	51 ± 17		
Sex			
Male		1388	46.17
Female		1617	53.79
Other		1	0.03
Education			
Low (e.g., without school-leaving qualification, lower secondary school)		231	7.68
Medium (e.g., high school diploma, secondary school)		1722	57.29
High (e.g., technical college, university)		1051	34.97
No answer		2	0.07
Professional status (multiple answers possible)			
Full-time employed		1042	34.66
Half-time employed		354	11.78
Reduced working hours		24	0.8
Self-employed		164	5.46
Student		204	6.79
Retired or pensioner		997	33.17
Unemployed		181	6.02
Other		100	3.33
No answer		16	0.53
Work in system-related job			
Yes		417	13.87
No		2,537	84.40
No answer		52	1.73
Marital status			
Married/registered partnership		1419	47.21
Widowed		113	3.76
Divorced or separated		344	11.44
Single		712	23.69
In a committed relationship, but not married		413	13.74
Other		5	0.17
Region			
Baden-Württemberg		350	11.64
Bayern		427	14.2
Berlin		175	5.82
Brandenburg		82	2.73
Bremen		26	0.86
Hamburg		93	3.09
Hessen		232	7.72
Mecklenburg-Vorpommern		65	2.16
Niedersachsen		254	8.45
Nordrhein-Westfalen		661	21.99
Rheinland-Pfalz		137	4.56
Saarland		40	1.33
Sachsen		186	6.19
Sachsen-Anhalt		83	2.76
Schleswig–Holstein		125	4.16
Thüringen		68	2.26
Monthly net income			
< 450 €		142	4.72
450–1000 €		347	11.54
1001–2000 €		915	30.44
2001–3000 €		729	24.25
3001–4000 €		398	13.24
> 4000 €		274	9.12
No answer		201	6.69

### Random parameter logit model

The model was analyzed using mixlogit command [40] in Stata 16.1. Table 3 shows the results of the RPL model. Five attributes (“excess mortality”, “individual risk of infection”, “decline in GDP”, “decrease in individual income”, and “curfews”) were included as random in the model, the attributes (“contact restrictions”, “closure of facilities”, “transmission of personal data”, and “mandatory masks in public”) entered the model as fixed. More information about the fixed and random parameter selection process can be found in the ESM Appendix.

**Table 3:** Results of the RPL model

Attributes	Levels	Mean		Se	[95%	Ci]	SD		Se	[95%	Ci]
Excess mortality	No excess mortality	1.12	***	0.03	1.06	1.18	0.95	***	0.04	0.87	1.02
	800 (+ 1%)	0.64	***	0.02	0.60	0.69	0.45	***	0.05	0.36	0.54
	4000 (+ 5%)	0.03		0.02	− 0.01	0.08	0.10		0.08	− 0.05	0.25
	8000 (+ 10%)	− 0.48	***	0.02	− 0.53	− 0.43	0.15	**	0.07	0.00	0.29
	16,000 (+ 20%)|24,000 (+ 30%)	− 1.32	***	0.04	− 1.40	− 1.24	− 1.65	***	0.12	− 1.88	− 1.41
Individual risk	No infection risk	0.86	***	0.03	0.81	0.91	0.75	***	0.04	0.67	0.82
of infection	5%	0.62	***	0.02	0.57	0.67	0.49	***	0.04	0.40	0.58
	10%	0.10	***	0.02	0.06	0.14	0.05		0.08	− 0.10	0.20
	15%	− 0.40	***	0.02	− 0.45	− 0.35	0.02		0.06	− 0.09	0.13
	25%|35%	− 1.18	***	0.04	− 1.25	− 1.11	− 1.30	***	0.11	− 1.51	− 1.09
Decline in GDP	No decline	0.54	***	0.02	0.50	0.58	− 0.34	***	0.04	− 0.43	− 0.25
	5% (2350 € pp)	0.36	***	0.02	0.32	0.40	− 0.07		0.08	− 0.24	0.09
	10% (4700 € pp)	0.03		0.02	− 0.01	0.07	− 0.02		0.05	− 0.12	0.08
	15% (7050 € pp)	− 0.27	***	0.02	− 0.32	− 0.23	− 0.04		0.04	− 0.13	0.04
	20% (9400 € pp)|25% (11,750 € pp)	− 0.65	***	0.03	− 0.71	− 0.60	0.47	***	0.13	0.22	0.72
Decrease in	No decrease	1.16	***	0.03	1.10	1.22	1.00	***	0.04	0.91	1.08
individual income	10%	0.91	***	0.02	0.86	0.95	− 0.14		0.10	− 0.34	0.05
	25%	0.19	***	0.02	0.15	0.24	0.04		0.07	− 0.09	0.18
	50%	− 0.61	***	0.03	− 0.67	− 0.56	0.03		0.06	− 0.09	0.15
	75|100%	− 1.64	***	0.04	− 1.73	− 1.56	− 0.93	***	0.18	− 1.27	− 0.58
Curfews	No curfews	0.06	***	0.02	0.02	0.10	0.28	***	0.05	0.17	0.38
	Closure of national borders	0.06	***	0.02	0.03	0.10	0.06		0.08	− 0.10	0.22
	Domestic travel restrictions	0.09	***	0.02	0.05	0.12	0.10	**	0.04	0.01	0.18
	Strict curfew	− 0.21	***	0.02	− 0.25	− 0.17	− 0.44	***	0.09	− 0.61	− 0.26
Contact restrictions	No restrictions	− 0.06	**	0.03	− 0.12	− 0.01					
	Max. 5 people	0.29	***	0.03	0.24	0.35					
	Max. 10 people	0.33	***	0.03	0.28	0.39					
	Max. 50 people	0.16	***	0.03	0.11	0.21					
	Max. 100 people	− 0.03		0.03	− 0.08	0.02					
	Max. 500 people	− 0.27	***	0.03	− 0.33	− 0.21					
	Max. 5000 people	− 0.43	***	0.03	− 0.48	− 0.37					
Closure of facilities	No closures	0.24	***	0.02	0.19	0.29					
	Kindergartens	− 0.23	***	0.02	− 0.28	− 0.18					
	Schools	− 0.14	***	0.03	− 0.19	− 0.09					
	Universities and colleges	− 0.05	*	0.03	− 0.10	0.00					
	Leisure and cultural activities	0.18	***	0.02	0.13	0.22					
	Non-system relevant businesses	0.00		0.02	− 0.05	0.05					
Transmission of	No transmission	0.11	***	0.02	0.07	0.14					
personal data	Health data	− 0.05	**	0.02	− 0.08	− 0.01					
	Contact data	− 0.01		0.02	− 0.05	0.03					
	Location data	− 0.05	***	0.02	− 0.09	− 0.01					
Mandatory masks	No mask requirement	− 0.35	***	0.02	− 0.39	− 0.31					
in public	Inside of buildings	0.13	***	0.02	0.10	0.17					
	Inside and outside of buildings	0.10	***	0.02	0.06	0.13					
	Public transportation	0.12	***	0.02	0.08	0.16					

Obs = 108,216; N = 3006; ll (null) =  − 32,103.63; ll (model (− 31,450.78; *df* = 55; AIC = 63,011.56; BIC = 63,539.12

*Mean* mean coefficient, *se* standard error; *ci* confidence interval, *SD* standard deviation, *pp* per person, *GDP* gross domestic product, *df* degrees of freedom, *AIC* Akaike Information Criterion, *BIC* = Bayesian Information Criterion

*p < *0.01*

**p < *0.1*

***p < *0.1*

The results include preference weight estimates (coefficient) for all attributes and standard deviation estimates for five attributes. All estimates are effects-coded. A positive sign reflects a positive preference for an attribute level, a negative sign a negative preference. Large coefficients indicate great impact on choice decisions, small coefficients indicate less impact on choice decisions. Similar for the standard deviations, large standard deviations indicate greater variability in preference weights, smaller standard deviations indicate smaller variability in preference weights.

The last levels of the first four attributes were varied for the scope test. For example, in the first questionnaire, the last level of the attribute “excess mortality” included “16,000 people (+ 20%)” and in the second questionnaire “24,000 people (+ 30%)”. The same was done with the last levels of the attributes “individual risk of infection”, “decline in GDP”, and “decrease in individual income”. The data from both questionnaires were aggregated for the main analysis.

As for the mean coefficients, most attribute levels were found to be statistically significant for all attributes. For four attributes, individual risk of infection, decrease in individual income, curfews, and mandatory masks in public, all levels were statistically significant. Almost all attribute levels thus had a statistically significant impact on choice decisions. The levels with the greatest positive influence and the greatest negative influence on respondents’ choice decisions both belong to the attribute “decrease in individual income”. The level with the greatest influence was “no decrease”, and the level with the greatest negative influence was “75|100%”. All levels of this attribute were statistically significantly different from each other and significantly different from zero.

Unobserved preference heterogeneity is shown by significant standard deviation of the mean coefficients. Standard deviations of the first and last levels of the random attributes are statistically significant suggesting large variation in preferences. For example, there were significant variations in preferences for no decrease in individual income and very high decrease in income.

**Fig. 1:**
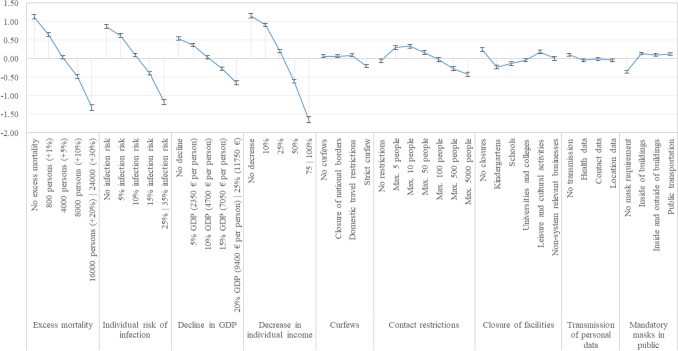
Preference weights in the random parameter logit model (95% confidence interval)

Figure 1 shows the graphical trend of the preference weights. Vertical distances between levels within an attribute and distances from the zero line indicate preference differences or indifferences. The greater the distance to zero, the more significant was the influence of the level on the choice decision of the respondents. Overlapping confidence intervals between levels within an attribute indicate indifferences between these levels. These levels are not statistically significantly different from each other, e.g., for the attribute “curfews” this applies to the first three levels. The levels “no curfews”, “closure of national borders”, and “domestic travel restrictions” are statistically significantly different from zero and thus have a significant influence on the choice decision. However, the confidence intervals of the levels overlap and are not statistically significantly different from each other.

### Relative attribute importance

**Fig. 2:**
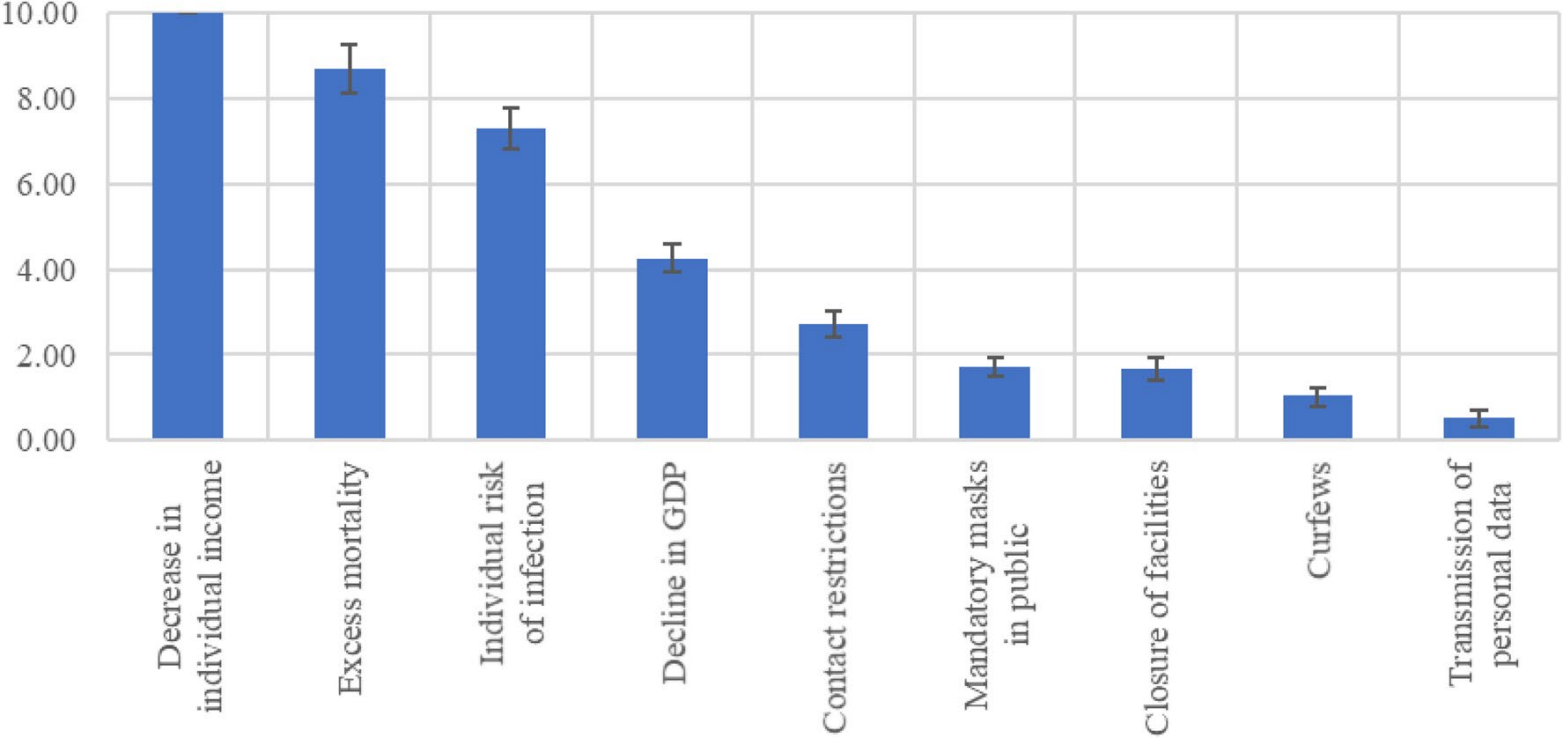
Relative attribute importance in descending order left to right from very important to less important (95% confidence interval)

The relative importance of the attributes was calculated based on the vertical distance between the most important and least important level within an attribute. The greater the difference, the more important the attribute. The importance is described by the change between the most important and least important level. The importance weights can be compared across different attributes. The calculated level differences were normalized on a 10-point scale. Figure 2 shows the relative importance for each attribute in descending order. The attribute “decrease in individual income” was the most important attribute closely followed by “excess mortality” and “individual risk of infection”. This is followed by “decline in GDP” and “contact restrictions” and then, equally, by the attributes “mandatory masks in public” and “closure of facilities”. The last two places are taken by the attributes “curfews” and “transmission of personal data”.

### Marginal rate of substitution

The marginal rate of substitution (MRS) measures trade-offs between two attributes, i.e., the relative impact of an attribute in a monetary metric as willingness to pay (WTP) or in an equivalent metric as willingness to accept (WTA) compensation for changes in a given attribute. It defines a respondent's willingness to substitute one good or item for another. The MRS is calculated as the ratio between two attribute coefficients of interest [21, 41]. In this study, a modified model was estimated to analyze the MRS with the attribute risk of infection as monetary equivalent. The risk attribute was included in the model as a linear continuous variable. The WTA of each attribute level was estimated by dividing the coefficient for each attribute level by the coefficient for the risk attribute. The results can be interpreted as the willingness of respondents to accept an additional risk of infection to avoid a specific event, such as a decrease in income. The results of the random parameter logit model used to calculate the WTA can be found in the ESM Appendix, as well as the WTA estimates generated using the marginal rate of substitution.

To simplify interpretation, the first level of each attribute was selected as the reference. The coefficient of the reference level was subtracted from the other level coefficients within an attribute. The results were plotted graphically in a diagram. Table 4 shows the WTA values for attributes excess mortality, GDP decrease, income decrease, and curfews. The values reflect the risk in percent that respondents would be willing to accept to avoid a worsening of the respective pandemic outcomes or interventions. Respondents would accept a greater risk of infection to avoid excess mortality and individual income decrease. Respondents would accept a risk of infection of about 37% to prevent a decrease in income from 0 to 75 respectively 100%. In comparison, respondents would accept a lower additional risk of infection to avoid GDP decrease, and curfews. Results for the remaining attributes are provided in Table 4 in the ESM Appendix.

**Table 4:** Willingness to accept risk of infection, in percent

	
	

The results of the separate models show that respondents in the scope model are willing to take a much greater risk of infection to prevent a decrease in income. As well, they would take a somewhat greater risk for the prevention of excess mortality and GDP decrease. See ESM Appendix.

### Best–worst scaling

In the BWS, the attributes “contact restrictions”, “closure of facilities”, and “curfews” were included, each with different time periods. The implementation of these interventions has probably more far-reaching effects on the economic, social and cultural life of the population and the country compared to mandatory masking and data transmission. In addition, the acceptance of the implementation of the individual interventions appears to be very time-dependent. Each of the attributes was assigned a period of 2, 4, and 12 weeks resulting in an overall of 42 choice items.

The results of the BWS are presented as standardized score for each item. First, the difference between the frequency of an item was chosen as best and the frequency of an item was chosen as worst was calculated. This difference was then divided by the number of times an item was available for choice across the “BWS-design”. The standardized scores indicate the relative impact of an item. The higher the score the more important the item is for the population.

**Fig. 3:**
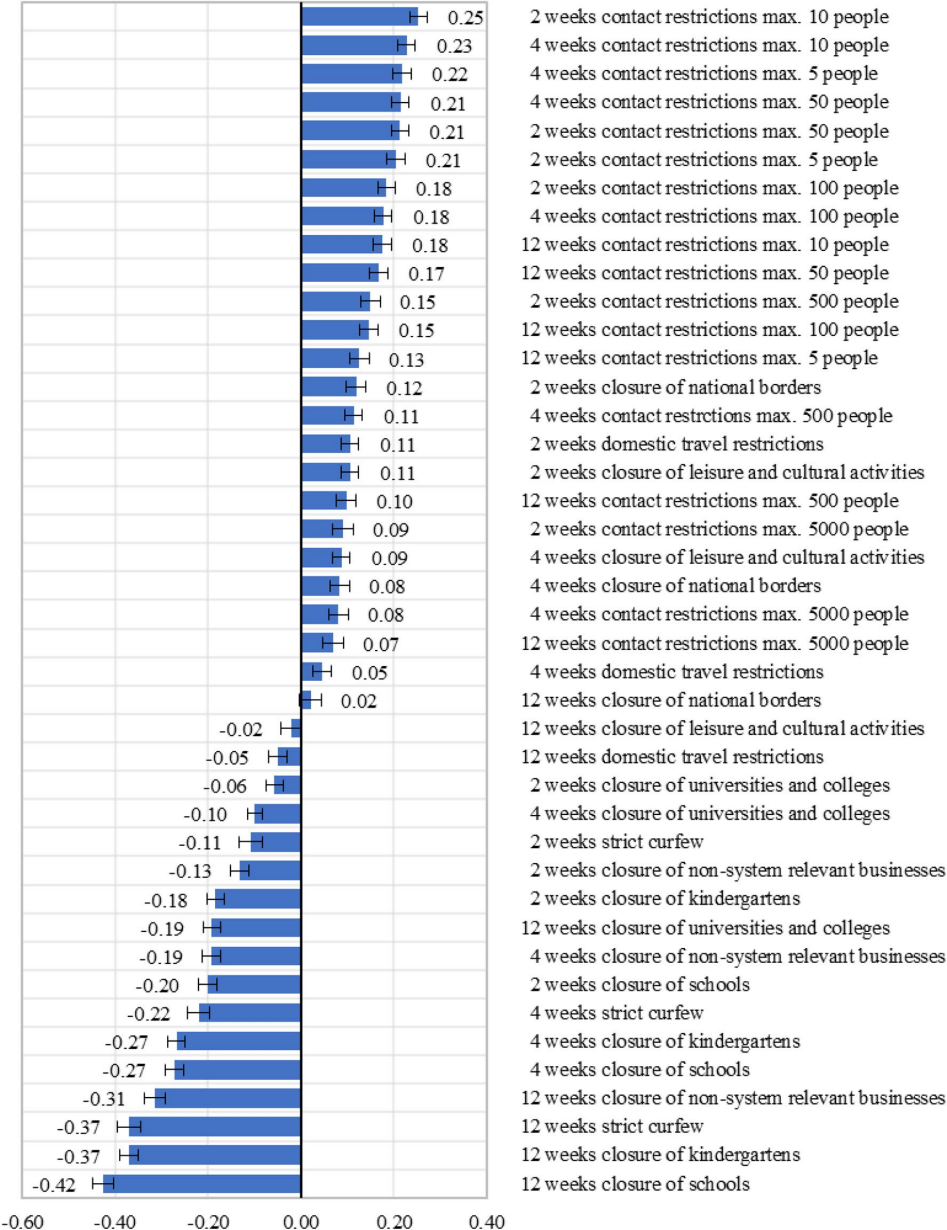
Standardized scores and 95% confidence intervals of items in the Best–worst scaling method

As in the DCE, contact constraints had the greatest impact on respondents and were thus most important. The scores of all contact restriction items have a positive sign (Fig. 3). A shorter period with a small number of maximum people allowed was preferred to a longer period. In contrast, closure of facilities and curfews were less frequently chosen as best items. The population showed a clear negative attitude with regard to a 12-week closure of schools and kindergartens and a strict curfew. The 12-week closure of schools was the least preferred intervention. The confidence intervals for this item are the only ones that do not overlap with any interval of any other item, indicating a significant difference.

## Discussion

The current global health crisis requires the engagement of experts from different disciplines, such as epidemiologists, virologists, economists, as well as the involvement of the population in the establishment of pandemic measures. Consideration of the population requires reliable measurement and interpretation of population preferences for risk–benefit trade-offs of the interventions to be established. This research study analyzes population preferences regarding measures to contain a viral pandemic and regarding potential consequences of these measures for economic, social, and cultural life. The result enables decision-makers to understand the preferences of the population and possible trade-offs that citizens are willing to make in a pandemic.

Many interesting and insightful preference studies have been conducted since the beginning of the corona pandemic. However, immediate, and long-term effects of non-pharmaceutical measures, as well as inferring which effects are consequences of which interventions, are difficult to model. This is evident in the different setups of preference studies on corona pandemic interventions and its potential effects on the economy and society. Decision context in preference studies ranges from mainly measure-related attributes and levels such as “stay-at-home orders” [42] to attributes and levels that represent exclusively pandemic indicators such as “number of new cases” [20]. Although some of the attributes and levels are similar to those used in other studies, the present study attempted to create a more holistic approach that considers interventions and effects of interventions equally. And the partial design not only reduced the size of the choice decisions to cognitively unburden respondents, but also took into account the fact that not all of the interventions specified need to be applied at the same time. A comparison with other preference studies is difficult not only because of the different decision models, but also because of the different pandemic incidence in the respective region. The results must be considered in relation to geographical and temporal survey factors. An advantage of our study is the large sample of 3000 respondents. This number is likely to be exceeded by only a few other studies and will be beneficial in further analyses.

The analysis showed that consequences of pandemic measures, such as excess mortality, risk of infection, decrease in income, and decrease in GDP had the most significant impact on respondents' choice decisions. The lower the expected consequences, the greater the impact of the corresponding attribute on choice decision in the DCE. Preventing individual income decrease had a greater importance compared to economic decrease. On the other hand, preventing excess mortality in the population was ranked as more important than lower individual risk of infection. But the most preferred measures may not necessarily be the most effective in practice. And conversely, the most effective measures in practice may not necessarily be the most popular in the population. Decision-makers need to find the right balance in establishing interventions. For this, non-pharmaceutical interventions need to be evaluated in terms of acceptability in in the population.

An effective non-pharmaceutical intervention is to limit gatherings of people. The attribute “contact restriction” had a significant impact on choice decisions in the aggregate model. Contact restrictions of a maximum of 5 to 10 persons were most preferred. A contact restriction to a maximum of 50 persons still had a positive but decreasing utility value. The threshold in the model appears to be a maximum of 100 persons. Another effective measures to control the pandemic is the closure of public facilities, such as schools and universities, as well non-essential businesses and curfews [31]. Our results show that, in general, no closures are preferred. Closure of kindergartens and schools was significantly rejected. However, respondents seemed indifferent to the closure of universities and non-essential businesses. Closures of leisure and cultural facilities, on the other hand, were favored. Curfews may have additional, although small, effects on controlling a pandemic. Strict curfews were clearly opposed by respondents in our study and had a significant negative impact on choice decisions.

The impact of masks on reducing virus transmission is difficult to estimate because measures were imposed mostly in combination rather than individually [29, 31]. Regarding effectiveness, studies show partially divergent results [43–45]. However, mandatory mask in public had a positive effect on choice decisions in our study. No mask requirement was clearly rejected. Other preference studies show similar results [4, 42]. Compared with the number of preference studies on vaccine acceptability, there appear to be fewer studies examining and evaluating the acceptability of non-pharmaceutical interventions in the general population [2, 21, 42, 46].

### Limitations

In terms of population representativeness of the sample, the lower educational groups were underrepresented, and the higher educational groups slightly overrepresented. There were differences from the average age of the German population. The age group 50 to 69 was overrepresented in the sample. This resulted in a higher average age compared to the actual average age of 44.5 years in Germany. When interpreting the results based on these study data and a possible transfer to the total population, this difference must be considered. In the RPL model, data from two questionnaires that differed in detail were aggregated. Separate analysis of the two questionnaires yielded nearly congruent result (see Appendix in the article last). Nevertheless, this approach is methodologically somewhat flawed.

The RPL model accounts for the heterogeneity of preferences. In this study, heterogeneous preferences were found in the sample. Further analysis was not performed and remains for subsequent studies.

### Further research

The influence of attributes on respondents' choice decisions defines the value of non-pharmaceutical interventions in the corona pandemic. Subgroup analysis such as latent class analysis (LCA) could be used to identify groups of respondents with different preferences in the sample. Subgroup-specific preference estimates, and segmentation results could be considered not only in economic models, such as cost-effectiveness analyses (CEAs) or multi-criteria decision analyses (MCDAs), but also in determining customized and appropriate pandemic interventions.

In addition, preferences are probably dependent on a time component. The BWS showed that shorter interventions are preferred over longer ones. Preferences could change over time as people gain experience in dealing with the pandemic. With restrictive continuation of pandemic measures, changes in preferences and acceptance in the population are conceivable. To analyze the influence of time, a repeated survey of the population would have to be conducted. Repeated surveys would reflect not only a possible change in the preferences over time and circumstances, but also the state of readiness of the population at different time points during the pandemic. Information about preferences and how they change over time would be critical for decision-makers in policy. Knowledge of how preferences change over time can be considered specifically in the implementation of pandemic measures and can promote and increase acceptance of these measures.

## Conclusion

This study should provide a rationale and reference point for further research on the acceptability of non-pharmaceutical interventions in the general population. To our knowledge, this is one of the few studies that analyzes preferences for non-pharmaceutical interventions using a comprehensive decision model that includes a variety of different pandemic measures and consequences. Restrictive pandemic measures can only be successful if measures are accepted by the population and if policy-makers can count on the approval of a large majority of citizens.

Complete control and containment of respiratory infections caused by coronaviruses seem illusive, but knowledge of population preferences may help to implement population-adapted and population-supported non-pharmaceutical interventions and prevent overloading of the health care system when needed.

## Electronic supplementary material

Below is the link to the electronic supplementary material.

Supplementary file 1 (PDF 888 KB)

## Appendix

**Table:**

#	Attribute	Level 1	Level 2	Level 3	Level 4	Level 5	Level 6	Level 7	SCOPE
1	Excess mortality	No excess mortality	800 people (+ 1%)	4000 people (+ 5%)	8000 people (+ 10%)	16,000 people (+ 20%)	–	–	24,000 People (+ 30%)
2	Individual risk of infection	No risk of infection	5%	10%	15%	25%	–	–	35%
3	Decline in gross domestic product (GDP)	No decline	− 5% (2350 € pp)	− 10% (4700 € pp)	− 15% (7050 € pp)	− 20% (9400 € pp)	–	–	− 25% (11,750 € pp)
4	Decrease in individual income	No decrease	− 10%	− 25%	− 50%	− 75%	–	–	-100%
5	Curfews	No curfews	Closure of national borders	Domestic travel restrictions	Strict curfew	–	–	–	–
6	Contact restrictions	No restrictions	Max. 5 people	Max. 10 people	Max. 50 people	Max. 100 people	Max. 500 people	Max. 5000 people	–
7	Closure of facilities	No closures	Kinder-gartens	Schools	Universities and colleges	Leisure and cultural activities	Non-system relevant businesses	–	–
8	Transmission of personal data	No transmission of data	Health data	Contact data	Location data	–	–	–	–
9	Mandatory masks in public	No mask requirement	Inside of buildings	Inside and outside of buildings	Public transportation	–	–	–	–
