# Supplementary material for: Population preferences for non-pharmaceutical interventions to control the SARS-CoV-2 pandemic: trade-offs among public health, individual rights, and economics
Source: Eur J Health Econ. 2022 Feb 9;23(9):1483–96. doi: 10.1007/s10198-022-01438-w (PMC9468277; doi:10.1007/s10198-022-01438-w)
Supplement: Supplementary file 1 — (PDF 888 KB) [file 10198_2022_1438_MOESM1_ESM.pdf]

Appendix

Discrete Choice Experiment

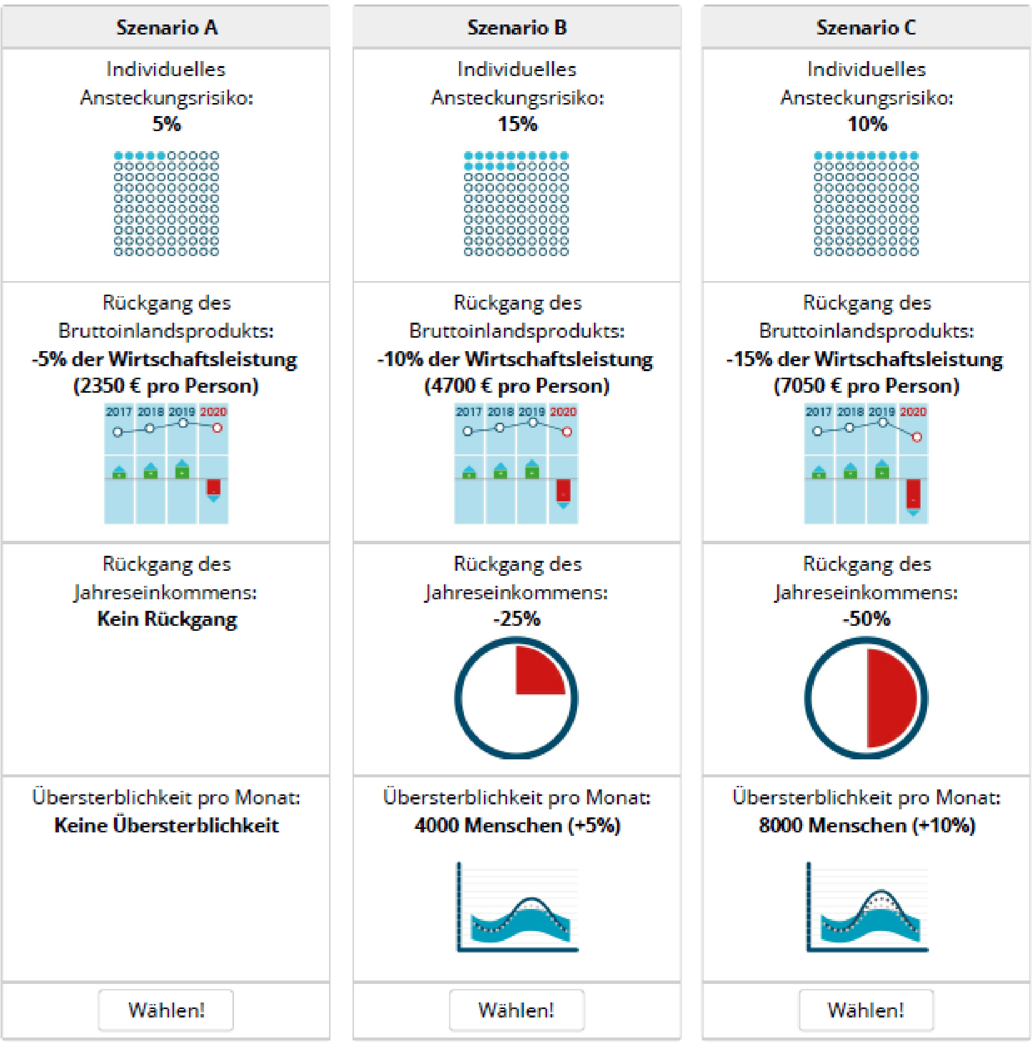

Figure 1: Example of a choice decision in the partial design. The question was: Which scenario would you prefer?

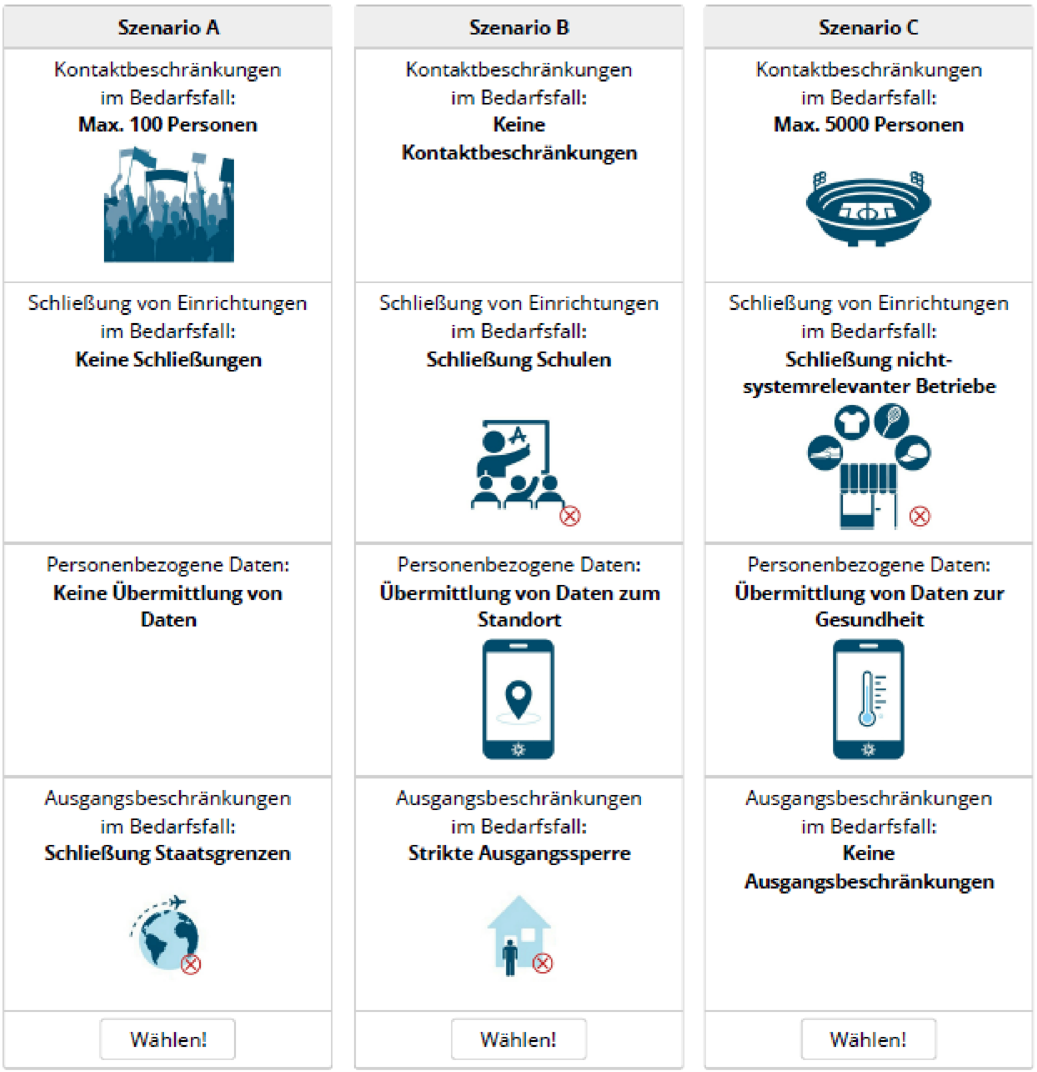

Figure 2: Choice task with varying attributes in the partial design.

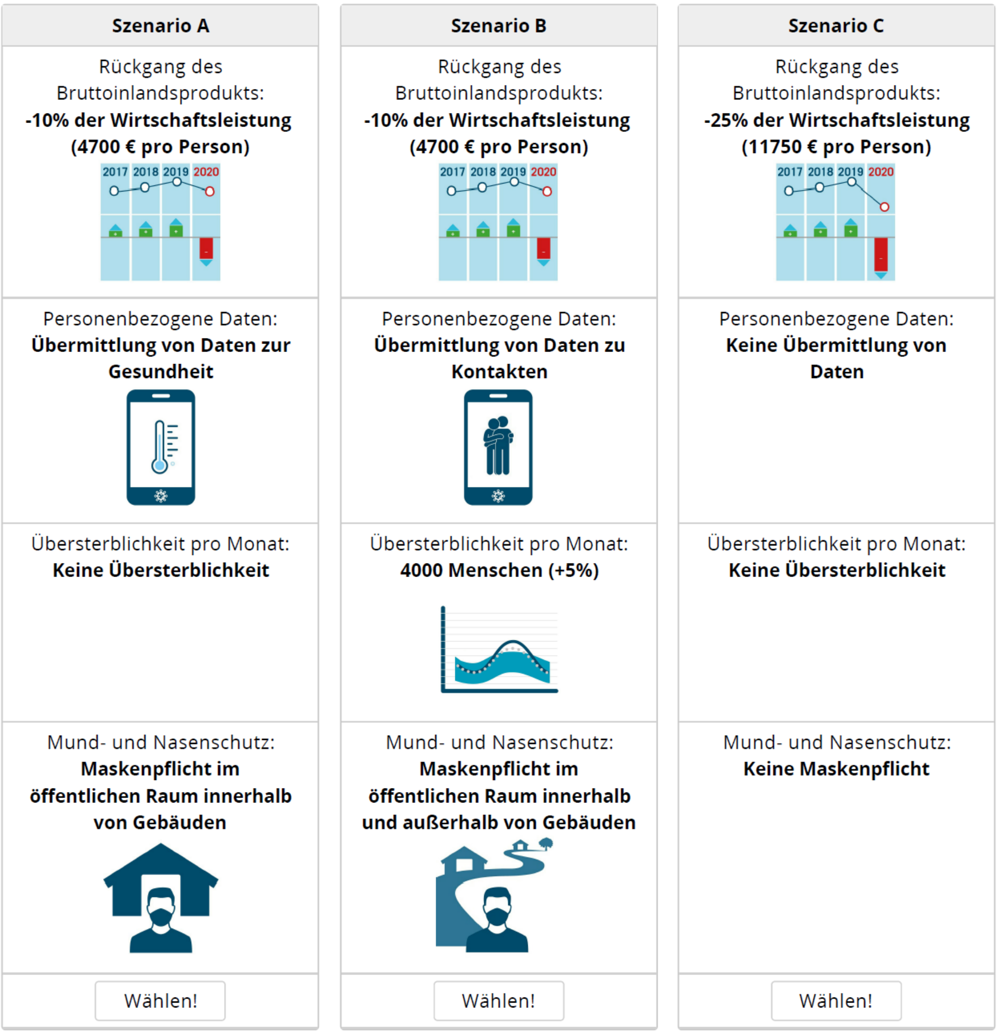

Figure 3: Choice task with scope test (25% decrease in GDP instead of 15%)

Selection of fixed and random parameters

The selection of fixed and random parameters was data-driven. Mixlogit command in Stata calculates models with a maximum of 20 dimensions (number of random parameters in the model). With this limitation and the extensive decision model of the study, not all variables could be set to random at the same time. Therefore, we systematically checked which attribute-level combination of fixed and random parameters yields the best model. To do this, we proceeded as follows. First, a calculation of all possible combinations with random variables was performed. This resulted in a total of 2^n = 2^9 = 512 -1 (minus 1 for a model with zero attributes) = 511 different attribute combinations for 9 attributes. Then, the total number of variables to be estimated in each combination was calculated. In the final calculation, only the models with no more than 20 random parameters in combination of fixed and random parameters were included. This resulted in a total of 333 models, which were successively estimated. Based on the model quality criteria AIC and BIC, the best of the 333 models was selected for the paper.

Model comparison – outcome of scope test

		Model 1 (N=1495)						Model 2 Scope (N=1511)					
**Attributes**	**Levels**	**Mean**	**se**	**p**	**SD**	**se**	**p**	**Mean**	**SE**	**se**	**SD**	**se**	**p**
Excess mortality	No excess mortality	1.13	0.04	0.00	0.97	0.06	0.00	1.12	0.04	0.00	0.94	0.05	0.00
	800 (+1%)	0.69	0.03	0.00	0.39	0.07	0.00	0.61	0.03	0.00	0.48	0.07	0.00
	4000 (+5%)	0.05	0.03	0.11	0.21	0.10	0.03	0.00	0.03	0.90	-0.14	0.08	0.08
	8000 (+10%)	-0.52	0.04	0.00	0.24	0.10	0.02	-0.44	0.03	0.00	0.05	0.10	0.61
	16000 (+20%) | **24000 (+30%)**	-1.35	0.06	0.00	-1.80	0.17	0.00	**-1.30**	**0.06**	**0.00**	**-1.34**	**0.16**	**0.00**
Individual risk of infection	No infection risk	0.83	0.04	0.00	0.76	0.05	0.00	0.87	0.04	0.00	0.71	0.06	0.00
	5%	0.64	0.03	0.00	0.45	0.06	0.00	0.61	0.03	0.00	0.38	0.09	0.00
	10%	0.07	0.03	0.05	-0.12	0.08	0.14	0.12	0.03	0.00	0.15	0.08	0.08
	15%	-0.45	0.04	0.00	0.08	0.07	0.24	-0.37	0.03	0.00	0.08	0.08	0.31
	25% | **35%**	-1.09	0.05	0.00	-1.18	0.13	0.00	**-1.23**	**0.05**	**0.00**	**-1.32**	**0.16**	**0.00**
Decline in GDP	No decline	0.54	0.03	0.00	-0.32	0.06	0.00	0.53	0.03	0.00	0.39	0.06	0.00
	5% (2350 € pp)	0.36	0.03	0.00	-0.01	0.08	0.90	0.36	0.03	0.00	-0.11	0.09	0.22
	10% (4700 € pp)	0.02	0.03	0.48	0.06	0.06	0.37	0.04	0.03	0.26	-0.10	0.07	0.18
	15% (7050 € pp)	-0.27	0.03	0.00	0.02	0.06	0.77	-0.28	0.03	0.00	0.02	0.07	0.82
	20% (9400 € pp) | **25% (11750 € pp)**	-0.65	0.04	0.00	0.26	0.15	0.08	**-0.65**	**0.04**	**0.00**	**-0.20**	**0.16**	**0.20**
Decrease in individual income	No decrease	1.11	0.04	0.00	0.98	0.06	0.00	1.22	0.04	0.00	1.00	0.06	0.00
	10%	0.93	0.03	0.00	-0.27	0.07	0.00	0.93	0.04	0.00	0.54	0.06	0.00
	25%	0.16	0.03	0.00	0.09	0.07	0.20	0.26	0.03	0.00	-0.06	0.09	0.50
	50%	-0.64	0.04	0.00	0.10	0.08	0.21	-0.57	0.04	0.00	0.03	0.08	0.67
	75% | **100%**	-1.55	0.06	0.00	-0.89	0.15	0.00	**-1.84**	**0.07**	**0.00**	**-1.50**	**0.15**	**0.00**
Curfews	No curfews	0.08	0.03	0.01	0.23	0.10	0.02	0.04	0.03	0.15	0.30	0.06	0.00
	Closure of national borders	0.06	0.03	0.04	-0.07	0.11	0.51	0.07	0.03	0.01	0.09	0.07	0.15
	Domestic travel restrictions	0.08	0.03	0.01	0.19	0.06	0.00	0.10	0.03	0.00	0.01	0.05	0.80
	Strict curfew	-0.21	0.03	0.00	-0.35	0.14	0.01	-0.21	0.03	0.00	-0.41	0.10	0.00
Contact restrictions	No restrictions	-0.07	0.04	0.06	.	.	.	-0.05	0.04	0.22	.	.	.
	Max. 5 people	0.34	0.04	0.00	.	.	.	0.26	0.04	0.00	.	.	.
	Max. 10 people	0.33	0.04	0.00	.	.	.	0.34	0.04	0.00	.	.	.
	Max. 50 people	0.14	0.04	0.00	.	.	.	0.18	0.04	0.00	.	.	.
	Max. 100 people	-0.02	0.04	0.56	.	.	.	-0.04	0.04	0.30	.	.	.
	Max. 500 people	-0.29	0.04	0.00	.	.	.	-0.25	0.04	0.00	.	.	.
	Max. 5000 people	-0.42	0.04	0.00	.	.	.	-0.44	0.04	0.00	.	.	.
Closure of facilities	No closures	0.21	0.03	0.00	.	.	.	0.27	0.03	0.00	.	.	.
	Kindergartens	-0.22	0.04	0.00	.	.	.	-0.24	0.04	0.00	.	.	.
	Schools	-0.15	0.04	0.00	.	.	.	-0.14	0.04	0.00	.	.	.
	Universities and colleges	-0.06	0.04	0.10	.	.	.	-0.02	0.04	0.49	.	.	.
	Leisure and cultural activities	0.20	0.03	0.00	.	.	.	0.15	0.03	0.00	.	.	.
	Non-system relevant businesses	0.02	0.04	0.63	.	.	.	-0.02	0.04	0.56	.	.	.
Transmission of personal data	No transmission	0.14	0.03	0.00	.	.	.	0.08	0.03	0.00	.	.	.
	Health data	-0.07	0.03	0.01	.	.	.	-0.02	0.03	0.35	.	.	.
	Contact data	0.00	0.03	0.91	.	.	.	-0.03	0.03	0.33	.	.	.
	Location data	-0.07	0.03	0.01	.	.	.	-0.03	0.03	0.29	.	.	.
Mandatory masks in public	No mask requirement	-0.38	0.03	0.00	.	.	.	-0.32	0.03	0.00	.	.	.
	Inside of buildings	0.15	0.03	0.00	.	.	.	0.12	0.03	0.00	.	.	.
	Inside and outside of buildings	0.11	0.03	0.00	.	.	.	0.09	0.03	0.00	.	.	.
	Public transportation	0.13	0.03	0.00	.	.	.	0.11	0.03	0.00	.	.	.
Observations		*53820*						*54396*					
N		*1495*						*1511*					
ll(null)		*-16017.06*						*-16060.36*					
ll(model)		*-15682.63*						*-15728.96*					
AIC		*31475.27*						*31567.93*					
BIC		*31964.40*						*32057.65*					

Table 1: Separate results of both models. The adjusted levels and the corresponding outcomes in the scope model are formatted in bold.

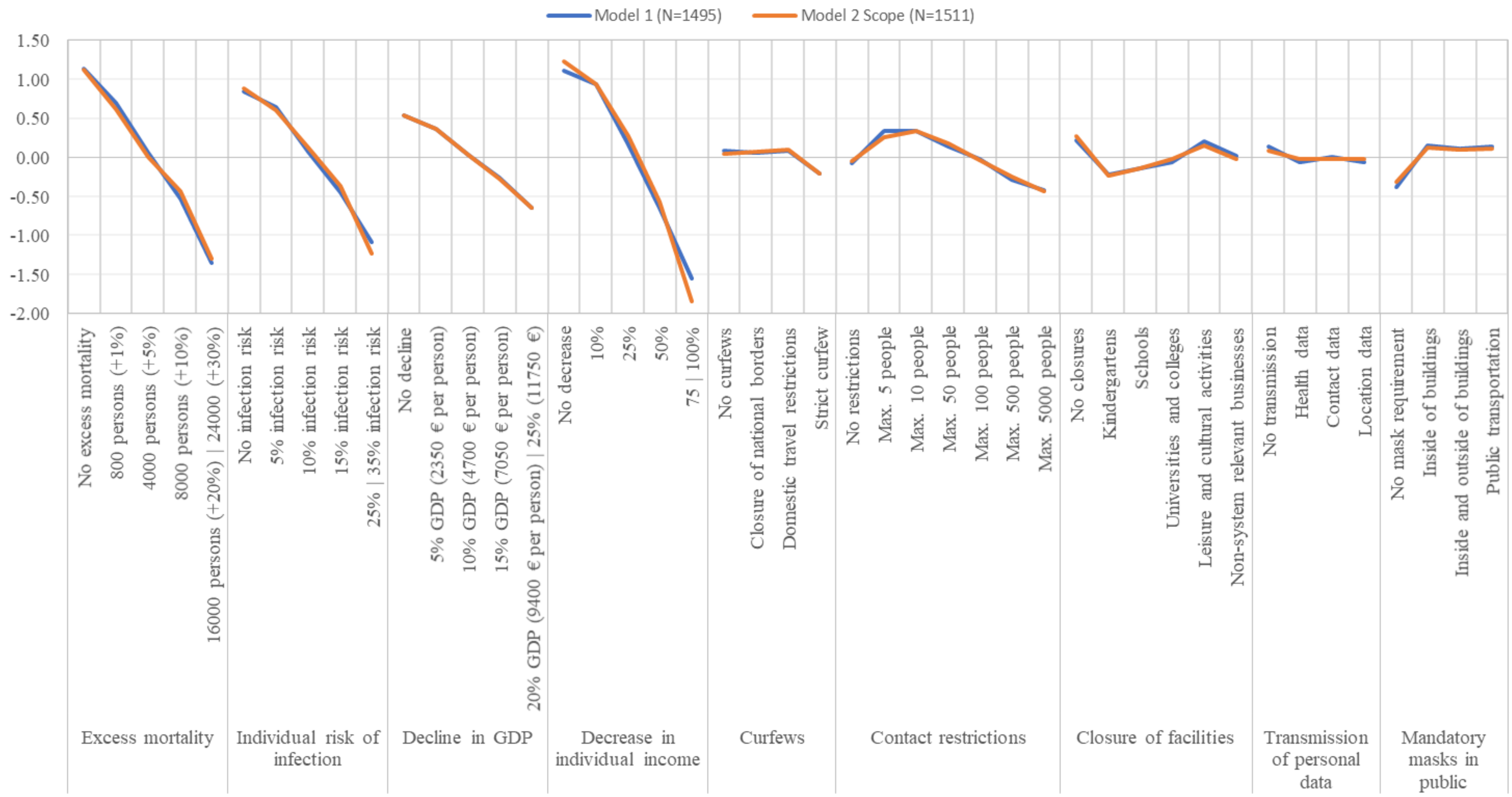

Figure 4: Chart comparison of the coefficients of both models

Marginal rate of substitution

Attributes	Levels	Mean	se	p	SD	se	p
Excess mortality	No excess mortality	1.150	0.030	0.000	0.945	0.039	0.000
	800 (+1%)	0.651	0.024	0.000	-0.463	0.045	0.000
	4000 (+5%)	0.024	0.024	0.300	0.102	0.070	0.145
	8000 (+10%)	-0.481	0.025	0.000	-0.098	0.075	0.192
	16000 (+20%) | 24000 (+30%)	-1.345	0.041	0.000	-0.486	0.129	0.000
Individual risk of infection		-0.077	0.002	0.000	0.064	0.002	0.000
Decline in GDP	No decline	0.547	0.022	0.000	0.323	0.049	0.000
	5% (2350 € pp)	0.356	0.022	0.000	0.240	0.052	0.000
	10% (4700 € pp)	0.036	0.022	0.107	0.007	0.061	0.904
	15% (7050 € pp)	-0.271	0.023	0.000	0.011	0.042	0.790
	20% (9400 € pp) | 25% (11750 € pp)	-0.668	0.027	0.000	-0.581	0.100	0.000
Decrease in individual income	No decrease	1.143	0.029	0.000	0.965	0.040	0.000
	10%	0.935	0.025	0.000	0.494	0.046	0.000
	25%	0.209	0.024	0.000	0.036	0.062	0.567
	50%	-0.609	0.027	0.000	-0.077	0.055	0.165
	75% | 100%	-1.679	0.043	0.000	-1.419	0.104	0.000
Curfews	No curfews	0.067	0.020	0.001	0.371	0.038	0.000
	Closure of national borders	0.062	0.019	0.001	0.228	0.044	0.000
	Domestic travel restrictions	0.092	0.019	0.000	0.070	0.058	0.224
	Strict curfew	-0.221	0.021	0.000	-0.670	0.073	0.000
Contact restrictions	No restrictions	-0.066	0.028	0.018	.	.	.
	Max. 5 people	0.299	0.027	0.000	.	.	.
	Max. 10 people	0.340	0.026	0.000	.	.	.
	Max. 50 people	0.160	0.027	0.000	.	.	.
	Max. 100 people	-0.034	0.028	0.214	.	.	.
	Max. 500 people	-0.271	0.028	0.000	.	.	.
	Max. 5000 people	-0.427	0.029	0.000	.	.	.
Closure of facilities	No closures	0.242	0.024	0.000	.	.	.
	Kindergartens	-0.229	0.025	0.000	.	.	.
	Schools	-0.145	0.025	0.000	.	.	.
	Universities and colleges	-0.042	0.025	0.094	.	.	.
	Leisure and cultural activities	0.182	0.024	0.000	.	.	.
	Non-system relevant businesses	-0.007	0.025	0.781	.	.	.
Transmission of personal data	No transmission	0.112	0.018	0.000	.	.	.
	Health data	-0.043	0.019	0.025	.	.	.
	Contact data	-0.014	0.019	0.461	.	.	.
	Location data	-0.055	0.019	0.004	.	.	.
Mandatory masks in public	No mask requirement	-0.343	0.020	0.000	.	.	.
	Inside of buildings	0.134	0.019	0.000	.	.	.
	Inside and outside of buildings	0.094	0.019	0.000	.	.	.
	Public transportation	0.116	0.019	0.000	.	.	.
Observations	*108,216*						
N	*3006*						
ll(null)	*-32189.26*						
ll(model)	*-31395.75*						
AIC	*62889.51*						
BIC	*63359.51*						

Table 2: Model with linear coded attribute "Individual risk of infection"

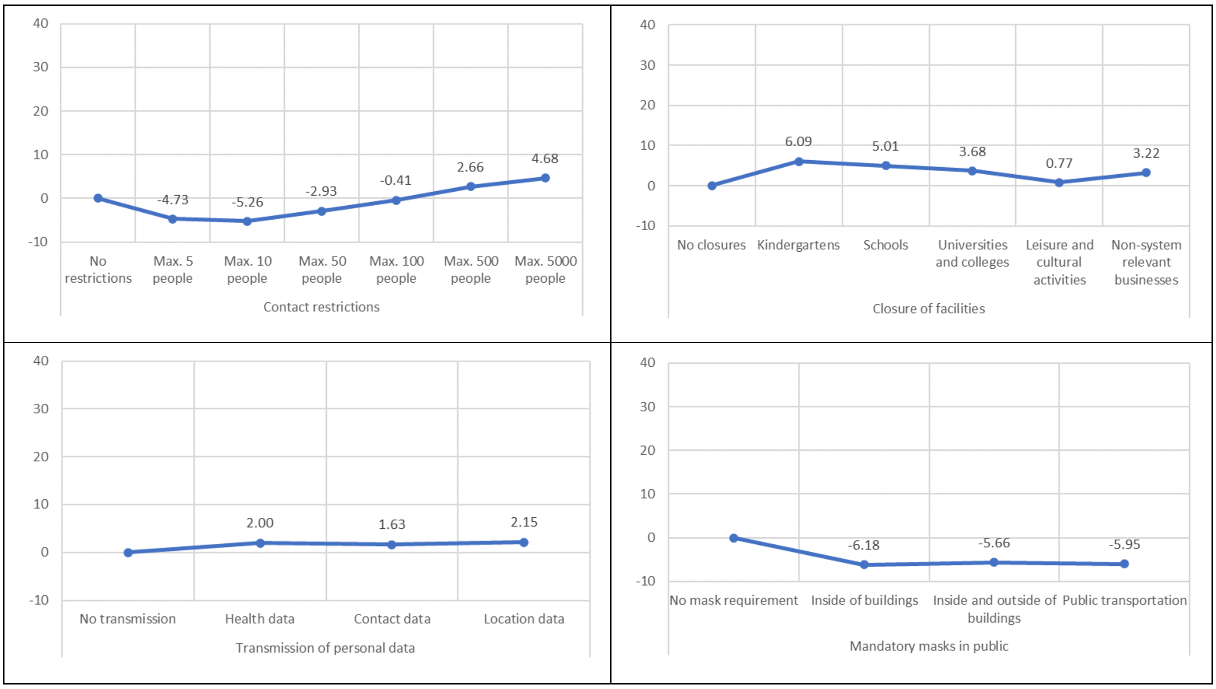

Table 3: Willingness to accept risk of infection, in percent

Sample interpretation: Respondents are willing to accept a 6% higher risk of infection if kindergartens are not closed. Reference level is "no closures". Respondents want to avoid the change from "no closures" to "closure of kindergartens".

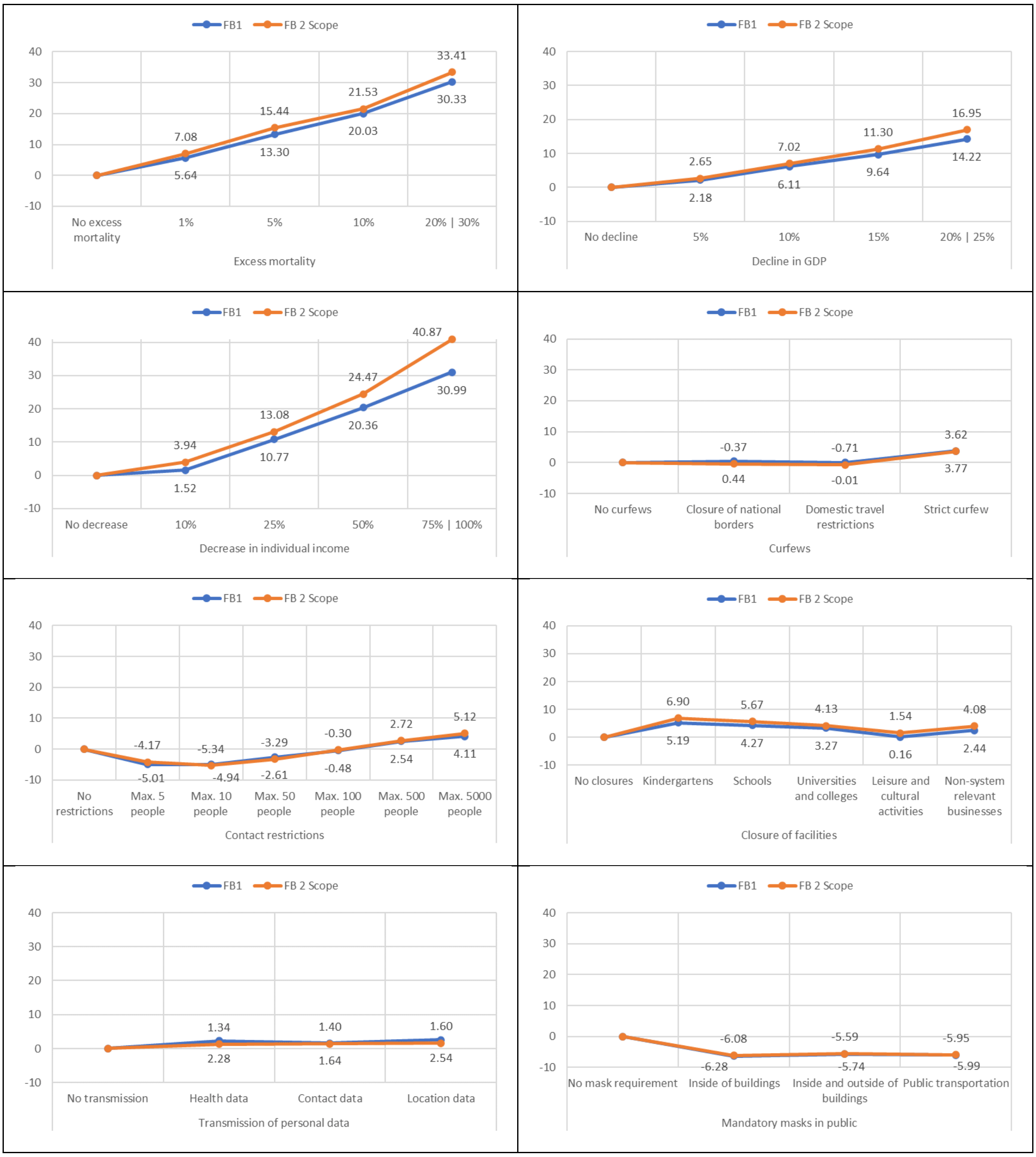

Table 4: Willingness to accept risk of infection in separate models, in percent

Best-Worst Scaling

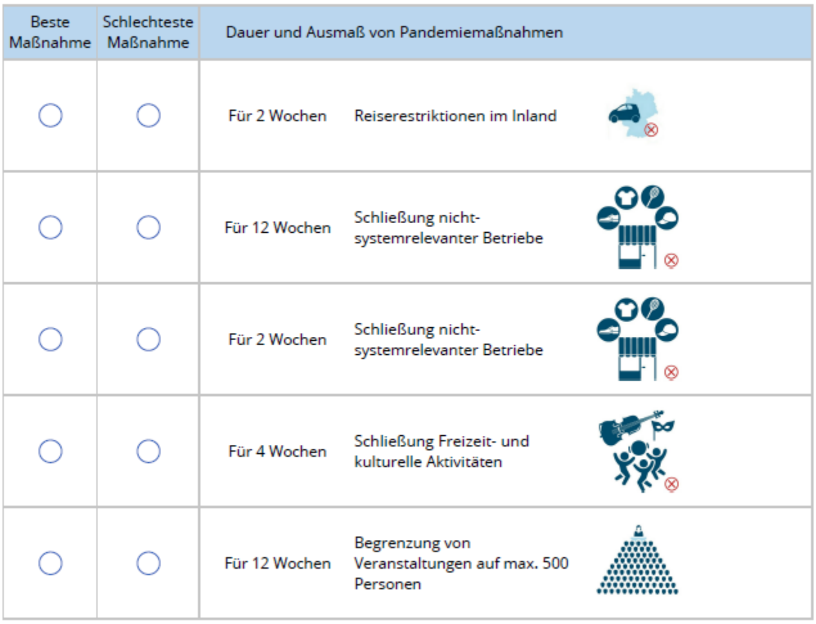

Beste Maßnahme	Schlechteste Maßnahme	Dauer und Ausmaß von Pandemiemaßnahmen	
◯	◯	Für 2 Wochen	Reiserestriktionen im Inland
◯	◯	Für 12 Wochen	Schließung nicht-systemrelevanter Betriebe
◯	◯	Für 2 Wochen	Schließung nicht-systemrelevanter Betriebe
◯	◯	Für 4 Wochen	Schließung Freizeit- und kulturelle Aktivitäten
◯	◯	Für 12 Wochen	Begrenzung von Veranstaltungen auf max. 500 Personen

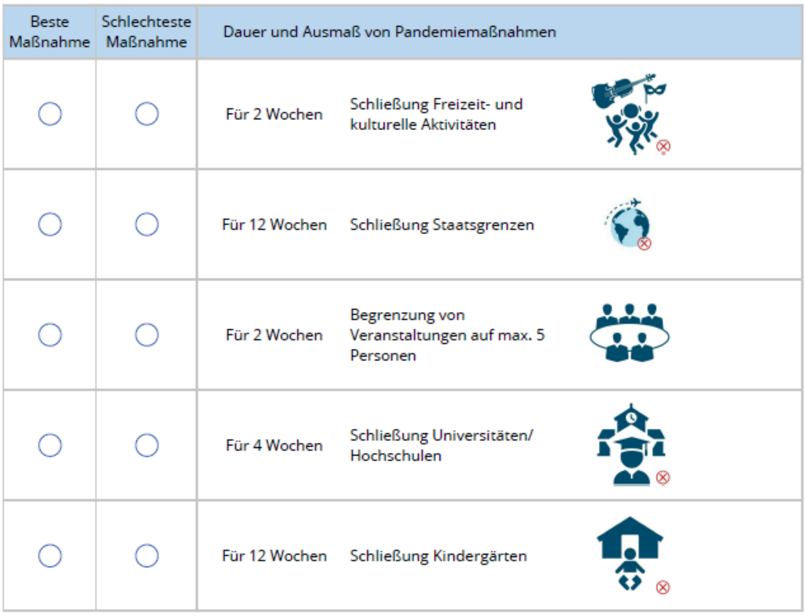

Beste Maßnahme	Schlechteste Maßnahme	Dauer und Ausmaß von Pandemiemaßnahmen	
◯	◯	Für 2 Wochen	Schließung Freizeit- und kulturelle Aktivitäten
◯	◯	Für 12 Wochen	Schließung Staatsgrenzen
◯	◯	Für 2 Wochen	Begrenzung von Veranstaltungen auf max. 5 Personen
◯	◯	Für 4 Wochen	Schließung Universitäten/ Hochschulen
◯	◯	Für 12 Wochen	Schließung Kindergärten

Figure 5: Screenshots of the Best-Worst Scaling exercise

Frequencies for the 42 items in the BWS selected as best and worst, the total number the items were shown, and the standardized Best-Worst Score.

Item	Label	Times Shown as Best	Times Selected as Best	Times Shown Worst	Times Selected as Worst	Best-Worst Score
1	2 weeks closure of national borders	3949	1021	3949	551	0.12
2	2 weeks domestic travel restrictions	3952	872	3952	456	0.11
3	2 weeks strict curfew	3937	866	3937	1290	-0.11
4	4 weeks closure of national borders	3937	982	3937	654	0.08
5	4 weeks domestic travel restrictions	3951	749	3951	570	0.05
6	4 weeks strict curfew	3940	670	3940	1538	-0.22
7	12 weeks closure of national borders	3949	949	3949	866	0.02
8	12 weeks domestic travel restrictions	3920	644	3920	839	-0.05
9	12 weeks strict curfew	3947	544	3947	2006	-0.37
10	2 weeks contact restrictions max. 5 people	3900	1172	3900	372	0.21
11	2 weeks contact restrictions max. 10 people	3950	1253	3950	257	0.25
12	2 weeks contact restrictions max. 50 people	3926	1139	3926	301	0.21
13	2 weeks contact restrictions max. 100 people	3953	1090	3953	363	0.18
14	2 weeks contact restrictions max. 500 people	3946	1052	3946	461	0.15
15	2 weeks contact restrictions max. 5000 people	3958	1049	3958	691	0.09
16	4 weeks contact restrictions max. 5 people	3933	1193	3933	341	0.22
17	4 weeks contact restrictions max. 10 people	3934	1172	3934	278	0.23
18	4 weeks contact restrictions max. 50 people	3948	1117	3948	272	0.21
19	4 weeks contact restrictions max. 100 people	3929	1020	3929	325	0.18
20	4 weeks contact restrctions max. 500 people	3938	892	3938	446	0.11
21	4 weeks contact restrictions max. 5000 people	3936	947	3936	630	0.08
22	12 weeks contact restrictions max. 5 people	3928	1083	3928	588	0.13
23	12 weeks contact restrictions max. 10 people	3931	1105	3931	415	0.18
24	12 weeks contact restrictions max. 50 people	3931	1072	3931	417	0.17
25	12 weeks contact restrictions max. 100 people	3915	1000	3915	429	0.15
26	12 weeks contact restrictions max. 500 people	3934	928	3934	546	0.10
27	12 weeks contact restrictions max. 5000 people	3956	999	3956	728	0.07
28	2 weeks closure of kindergartens	3940	346	3940	1071	-0.18
29	2 weeks closure of schools	3923	387	3923	1173	-0.20
30	2 weeks closure of universities and colleges	3927	447	3927	669	-0.06
31	2 weeks closure of leisure and cultural activities	3930	883	3930	470	0.11
32	2 weeks closure of non-system relevant businesses	3950	468	3950	985	-0.13
33	4 weeks closure of kindergartens	3930	209	3930	1260	-0.27
34	4 weeks closure of schools	3954	328	3954	1403	-0.27
35	4 weeks closure of universities and colleges	3931	309	3931	698	-0.10
36	4 weeks closure of leisure and cultural activities	3937	827	3937	482	0.09
37	4 weeks closure of non-system relevant businesses	3923	396	3923	1153	-0.19
38	12 weeks closure of kindergartens	3909	200	3909	1648	-0.37
39	12 weeks closure of schools	3919	285	3919	1949	-0.42
40	12 weeks closure of universities and colleges	3952	293	3952	1052	-0.19
41	12 weeks closure of leisure and cultural activities	3959	750	3959	834	-0.02
42	12 weeks closure of non-system relevant businesses	3918	358	3918	1589	-0.31

Table 5: Frequencies of items in the BWS exercise
